# Urinary interleukin-9 in youth with type 1 diabetes mellitus

**DOI:** 10.1007/s00592-022-01873-4

**Published:** 2022-04-20

**Authors:** Julie Semenchuk, Katie Sullivan, Rahim Moineddin, Farid Mahmud, Allison Dart, Brandy Wicklow, Fengxia Xiao, Thalia Medeiros, James Scholey, Dylan Burger

**Affiliations:** 1 Department of Medicine, Toronto General Hospital, University Health Network, University of Toronto, 200 Elizabeth Street, Toronto, ON M5G 2C4 Canada; 2 Department of Medicine, Renal and Hypertension Division, University of Pennsylvania, Philadelphia, USA; 3 Department of Family and Community Medicine, University of Toronto, Toronto, Canada; 4 Department of Pediatrics, The Hospital for Sick Children, University of Toronto, Toronto, Canada; 5 Department of Pediatrics and Child Health, Children’s Hospital Research Institute of Manitoba, Diabetes Research Envisioned and Accomplished in Manitoba Research Team, University of Manitoba, Winnipeg, Canada; 6 Department of Medicine, Kidney Research Centre, Ottawa Hospital Research Institute, University of Ottawa, Ottawa, Canada; 7 Department of Physiology, University of Toronto, Toronto, Canada

**Keywords:** Cytokines, Albuminuria, IL9, Extracellular vesicle, Podocyte, Type 1 diabetes mellitus

## Abstract

**Aims:**

Interleukin-9 (IL-9) attenuates podocyte injury in experimental kidney disease, but its role in diabetic nephropathy is unknown. We sought to relate urinary IL-9 levels to the release of podocyte-derived extracellular vesicles (EVs) in youth with type 1 diabetes. We related urinary IL-9 levels to clinical variables and studied interactions between urinary IL-9, vascular endothelial growth factor (VEGF), tumor necrosis factor alpha (TNFα) and interleukin-6 (IL-6) on urinary albumin/creatinine ratio (ACR) a functional measure of podocyte injury.

**Methods:**

We performed an analysis of urine samples and clinical data from a cohort of youth with type 1 diabetes (*n* = 53). Cytokines were measured using a Luminex platform (Eve Technologies), and nanoscale flow cytometry was employed to quantify urinary podocyte-derived EVs. All urinary measures were normalized to urinary creatinine.

**Results:**

Mean age was 14.7 ± 1.6 years, and the mean time from diagnosis was 6.7 ± 2.9 years. Mean HbA1c was 70.3 ± 13.9 mmol/mol, mean ACR was 1.3 ± 1.9 mg/mmol, and mean eGFR was 140.3 ± 32.6 ml/min/1.73 m^2^. IL-9 was inversely related to podocyte EVs (*r* = − 0.56, *p* = 0.003). IL-9 was also inversely related to blood glucose, HbA1C and eGFR (*r* = − 0.44, *p* = 0.002; *r* = − 0.41, *p* = 0.003; *r* = − 0.49, *p* < 0.001, respectively) and positively correlated with systolic BP (*r* = 0.30, *p* = 0.04). There was a significant interaction between IL-9, EVs and ACR (*p* = 0.0143), and the relationship between IL-9 and ACR depended on VEGF (*p* = 0.0083), TNFα (*p* = 0.0231) and IL-6 levels (*p* = 0.0178).

**Conclusions:**

IL-9 is associated with podocyte injury in early type 1 diabetes, and there are complex interactions between urinary IL-9, inflammatory cytokines and ACR.

## Introduction

Interleukin-9 (IL-9) is a T cell-derived cytokine, generally felt to be pro-inflammatory, which has been implicated in the regulation of both innate and adaptive immunity. [1, 2] Clinically, elevated serum IL-9 levels have been observed in a number of diseases including systemic lupus erythematosus [3], asthma [4] and coronary artery disease [5]. In a recent study of experimental murine kidney disease, Xiong and coworkers reported that intra-renal IL-9 attenuated podocyte injury in mice with adriamycin-induced nephropathy [6]. IL-9 knockout mice exhibited more podocyte injury and loss of podocyte number in comparison with wildtype mice [6]. The impact of IL-9 on podocyte injury in human kidney disease remains largely unexplored.

Mesangial matrix expansion is a dominant feature of early kidney disease in type 1 diabetes mellitus (T1D), particularly in relationship to hyperfiltration and early declines in glomerular filtration rate (GFR) [7]. Ultrastructural abnormalities in glomerular podocytes also occur in T1D although the changes are subtle, especially in early kidney disease [8]. A decrease in glomerular podocyte number occurs in the Akita mouse, an experimental model of T1D, and the decrease precedes any rise in the urinary albumin excretion rate [9]. These findings suggest that early podocyte injury is an important feature of diabetic kidney disease and that podocyte injury may contribute to functional changes like a rise in albumin excretion. Identifying biomarkers that correlate with early podocyte injury may help risk stratify patients early in the course of their disease.

Extracellular vesicles (EVs), including microparticles, exosomes, and apoptotic bodies, are cell-membrane-derived vesicles released by a variety of cell types in response to injury [10–12]. Cultured podocytes release EVs when subjected to mechanical strain or high glucose concentrations [13]. In accord with these in vitro observations, podocyte-derived EVs are also present in the urine of diabetic mice, and we found increased podocyte-derived EVs in the urine of adults with T1D [13, 14]. More recently, we reported that there was a significant correlation between podocyte-derived EVs and both blood glucose levels and eGFR in youth with TID [15]. However, the relationship between podocyte-derived EVs and IL-9 in T1D is unknown.

Accordingly, our aim in this exploratory study was to examine the relationship between urinary IL-9 and urinary podocyte-derived EVs in a well-characterized cohort of youth with T1D. Our hypothesis was that higher levels of IL-9 would be associated with lower podocyte-derived EV numbers in the urine, reflecting less podocyte injury and a protective effect of IL-9. We evaluated the relationships between IL-9 and HbA1C, blood glucose (BG), eGFR and blood pressure (BP). We hypothesized there would be negative correlations with each, supporting a reno-protective effect. In an exploratory analysis, we also studied interactions between IL-9, the albumin/creatinine ratio (ACR) and urinary cytokines linked to the progression of diabetic nephropathy: vascular endothelial growth factor (VEGF), interleukin-6 (IL-6) and tumor necrosis factor alpha (TNFα). [16–18].

## Methods

### Ethical statement and cohort description

We examined urine samples from a Cohort created from Canadian participants recruited from the observational arm of the Adolescent Type 1 Diabetes Cardio-Renal Intervention Trial (AdDIT) [19]. In brief, eligible individuals were 10 to 16 years of age with a diagnosis of Type 1 diabetes. The size of the cohort was based on the exploratory analysis that showed significant relationships between EVs and the clinical variables BG and eGFR [15]. Exclusion criteria included other forms of diabetes, pregnancy or unwillingness to adhere to contraceptive advice and pregnancy testing, severe hyperlipidemia or a family history suggesting familial hypercholesterolemia, the presence of coexisting conditions (excluding treated hypothyroidism and celiac disease), proliferative retinopathy, and the presence of non-diabetic renal disease. Study participants and their guardians gave informed consent. Studies were approved by The Hospital for Sick Children, Research Ethics Board at the University of Toronto (ID# 1,000,012,240).

### Serum and urine samples

Fasting first morning midstream urine samples were collected for all participants in the cohort. Urine samples were centrifuged at 2000 g at 4 °C for 6 min before storage in −80 °C. All analyses were conducted from a single urine sample from each participant. A single venous blood sample was also taken at the time of urine collection for the analysis of blood glucose levels and HbA1c. HbA1c was measured by enzymatic assay (Architect Analyzer; Abbott Diagnostics, USA).

### Urinary cytokines

Urinary levels of cytokines/chemokines were measured with Cytokine/Chemokine Panel Luminex Assay [19] from the urine samples. The urine samples were centrifuged at 1500 g for 15 min, separated into 1 ml aliquots and frozen at − 80 °C. The urine was then thawed at 4 °C one day prior to use [19]. We limited our cytokine/chemokine analysis to IL-9, VEGF, TNFα and IL-6 to maintain statistical power. The accuracy and precision of the urinary cytokine/chemokine assay is available from the vendor at http://www.millipore.com/userguides/tech1/proto_mpxhcyto-60k. We have previously published the detection limits of the assays. [20].

### Podocyte extracellular vesicles

Quantification of podocyte EVs was performed using our previously described nanoscale flow cytometry approach [13–15]. Briefly, stored urine samples were thawed, and EVs were isolated by sequential centrifugation first 2500 × g for 10 min at 4 °C, followed by 20,000 × g for 20 min at 4 °C. [9] The pellet was re-suspended in Annexin V Binding buffer with Annexin V FITC (1:50, Biolegend, California, USA). Podocyte origin was confirmed by co-staining with an anti-podoplanin APC-conjugated antibody (1:100, Biolegend, California, USA). Samples were analyzed by nanoscale flow cytometry (CytoFLEX S, Beckman Coulter, USA), and ApogeeMix beads (Cat. 1493–Apogee Flow Systems, UK) were used for size calibration. EVs were defined as particles sized between ~ 100–1000 nm. Samples labeled with isotype controls, antibodies alone in buffer, and unlabeled samples were analyzed as controls. FlowJo ver 7.6.5 was used for analysis. Levels of urinary EVs were normalized to urinary creatinine levels and podocyte EV levels were expressed as number/umolCr.

### ACR measurement

ACR was measured in 2 sets of 3 of the collected urine samples. A normal albumin excretion rate was defined this as an ACR < 2.0 mg/mmol [21]. Microalbuminuria was defined as an ACR of 2.0 to 20 mg/mmol.

### Estimated glomerular filtration rate (eGFR)

eGFR was calculated using the Larsson equation (cystatin C) as previously described [19]. Serum cystatin C was measured by a single operator using an immunoassay (Dade Behring Diagnostics, Newark, DE, USA) conducted on a BN Prospec System nephelometer [19]. Hyperfiltration was defined as an eGFR > 135 ml/min/1.73m2. [19].

### Blood pressure

BP values were the mean of two separate in-clinic measurements using an automated and validated BP cuff. Normal BP, elevated BP, stage one and stage two hypertension were defined as per American Academy of Pediatric Guidelines [22]. We calculated the BP percentiles using (https://apps.cpeg-gcep.net/BPz_cpeg/., accessed July 2020). [15].

### Statistical analysis

Descriptive statistics were used to describe the clinical characteristics of the sample, including mean and standard deviation for continuous measures, frequency and percentages for categorical variables. Spearman correlations were used to assess the strength of the association among continuous measures. In all parts of the analysis, *p* values were corrected for multiple comparisons using false discovery rate method. Our primary outcome of interest was the relationship between IL-9 and podocyte EVs. Data were disaggregated by sex to confirm the relationship. Secondary outcomes included the relationship between IL-9 and BP, glycemic control, eGFR and the cytokines VEGF, TNFα and IL-6. We then looked for interactions between IL-9, podocyte EVs and ACR. Finally, we looked for interactions between IL-9, the cytokines VEGF, TNFα and IL-6, and ACR. We log-transformed the continuous measures and used linear regression to assess the significance of the interactions.

## Results

### Clinical characteristics of the study cohort

The mean age was 14.7 ± 1.6 years, and the mean duration of time from diagnosis of diabetes was 6.7 ± 2.9 years. There were 26 males and 27 females. Ethnicity of the T1D group was as follows: 32/53 (60.4%) were white, 6/53 (11.3%) were black, 3/53 (5.7%) were South Asian, 4/53 (7.5%) were South East Asian, and 8/53 (15.1%) were classified as other. HbA1C levels averaged 70.3(8.6%) ± 13.9(3.1%) mmol/mol (Table 1), and the average BMI (kg/m2) was 22.1 ± 3.7 kg/m2. The mean eGFR (Larsson) was 140.3 ± 32.6 ml/min/1.73m2, and 47% of the cohort exhibited hyperfiltration. The mean ACR was 1.3 ± 1.9. Only 13% of the cohort exhibited an ACR > 2 mg/mmol (Table 1).

**Table 1:** Clinical characteristics of the study cohort

Clinical characteristic	Youth with type 1 DM
*N* = 53	
Age(years)	14.7 ± 1.6
Sex, male, *N* (%)	26 (49)
Ethnicity, *N* (%)	
White	32 (62.3)
Black	6 (11.3)
South Asian	3 (5.7)
South East Asian	4 (7.5)
Other	8 (15.1)
Glycated hemoglobin, HbA1c mmol/mol	70.3 ± 13.9
HbA1C %	8.6 ± 3.1
Diabetes duration (years)	6.7 ± 2.9
Body mass index, BMI (kg/m2)	22.1 ± 3.7
Z-score BMI	0.6 ± 1.0
Systolic blood pressure, SBP (mmHg)	114.6 ± 9.6
Diastolic blood pressure, DBP (mmHg)	67.2 ± 6.6
Blood pressure classification N (%)	
Normal	35 (63)
Elevated	14 (26.4)
Albumin/creatinine ratio, ACR (mg/mmol)	1.3 ± 1.9
% Microalbuminuria (> 2 mg/mmol)	13.2% (7/53)
Estimated glomerular filtration rate, eGFR	140.3 ± 32.6
% Hyperfiltration > 135	47% (25/53)

Values are expressed as mean ± SD for normally distributed variables or as median (minimum–maximum) for non-normally distributed. Microalbuminuria was defined as ACR > 2 mg/mmol. Hyperfiltration was defined as eGFR > 135 ml min/1.73 m^2^. The fraction of individuals with hyperfiltration is a percentage of the total number of subjects. *DBP* diastolic blood pressure; *SBP* systolic blood pressure

### Urinary IL-9 and urinary podocyte extracellular vesicles

**Fig. 1:**
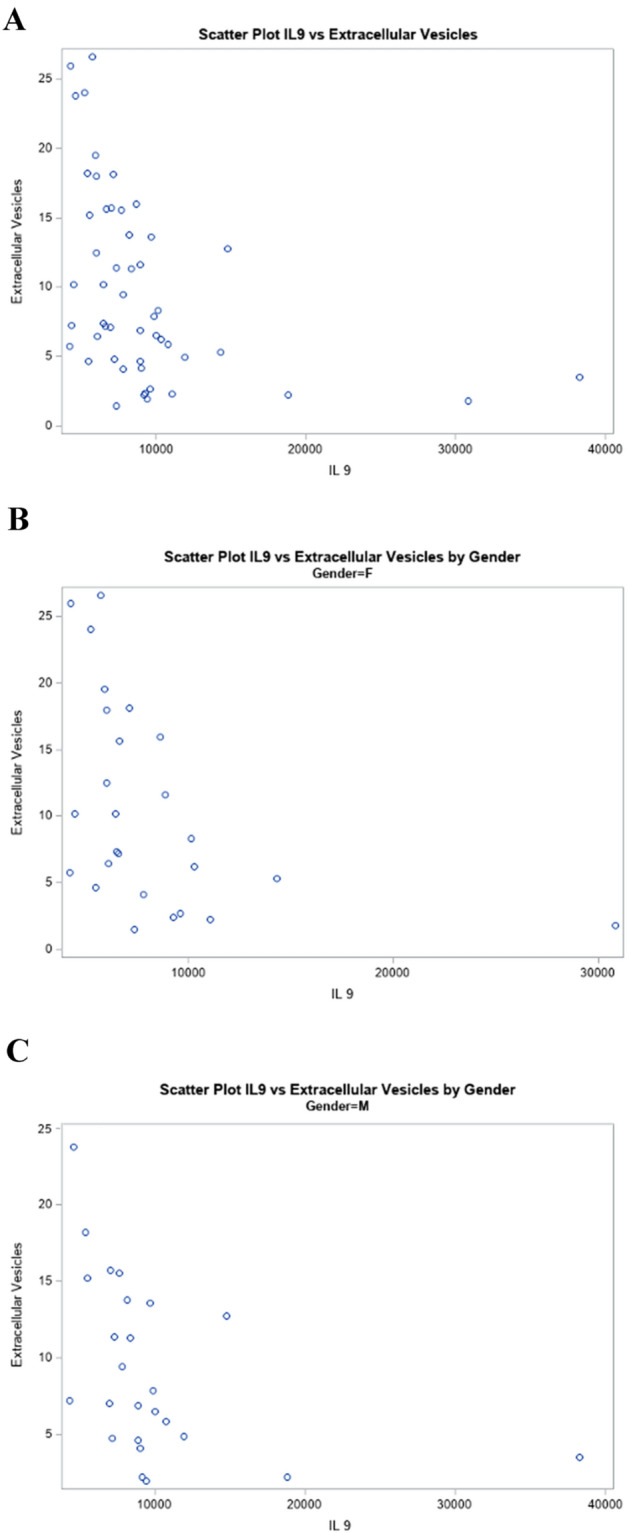
Scatter plots of the relationship between urinary interleukin-9 (IL-9) and podocyte-derived extracellular vesicles (EVs) in adolescents with T1D: **A** all subjects (*N* = 53) (*r* = −0.55692, *p* < 0.0003); and **B** females (*N* = 27) (*r* = −0.55692, *p* = 0.0031); and **C** males (*N* = 26) (*r* = −0.58154, *p* = 0.0023)

The median value for the podocyte-derived EVs normalized to urinary creatinine (EV/UCr) was 7.88 in the cohort, and as we previously reported there was no differences in the median urinary EV values between male and female subjects [15]. There was a negative correlation between IL-9 and EV (*r* = −0.56, *p* < 0.0003) such that higher values of IL-9 were associated with lower EV shown in Fig. 1A. The relationship was similar in female (*r* = −0.56, *p* = 0.003) and male subjects (*r* = −0.58, *p* = 0.002) as seen in Fig. 1B and C.

### Spearman correlation coefficients relating urinary IL-9, clinical variables and the cytokines VEGF, TNFα and IL-6

There was a negative correlation between IL-9 and eGFR (*r* = − 0.49, *p* < 0.0005) such that higher values of eGFR were associated with lower IL-9 levels (Table 2). There was a negative correlation between IL-9 and BG (*r* = − 0.44, *p* < 0.002) such that higher BG values were associated with lower IL-9 levels. Similarly, there was a negative correlation between IL-9 and HbA1C (*r* = − 0.41, *p* < 0.004) with higher HbA1C measurements associated with lower IL-9 levels. In contrast, there was a positive correlation between IL-9 and systolic blood pressure (SBP) (*r* = 0.30, *p* < 0.04) but not for IL-9 and diastolic blood pressure (DBP) (*r* = 0.05, *p* = 0.76).

**Table 2:** Spearman correlations coefficients for observed relationships with urinary interleukin-9

Clinical variable	Spearman correlation	*p* value
eGFR	− 0.49401	0.0005
Blood glucose	− 0.43534	0.0023
ACR	− 0.2601	0.0752
Systolic blood pressure	0.29846	0.0417
Diastolic blood pressure	0.04467	0.7556
HbA1C	− 0.40896	0.0041
Podocyte EV	− 0.55692	0.0003
Cytokines		
VEGF	0.71701	0.0003
TNFα	0.55391	0.0003
IL-6	0.46874	0.0014

*eGFR* estimated glomerular filtration rate, *ACR* albumin/creatinine ratio, *EV* extracellular vesicle, *VEGF* vascular endothelial growth factor, *TNFα* tumor necrosis factor alpha, *IL-6* interleukin-6

There were positive correlations between IL-9 and VEGF (*r* = 0.72, *p* < 0.0003), IL-9 and TNFα (*r* = 0.55, *p* < 0.0003) and IL-9 and IL-6 (*r* = 0.47, *p* = 0.001).

### Spearman correlation coefficients relating urinary IL-9, EV and ACR

**Fig. 2:**
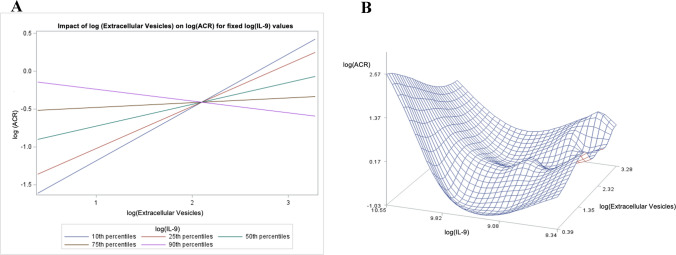
Interactions between urinary podocyte-derived extracellular vesicles (EVs), interleukin-9 (IL-9) and the albumin/creatinine ratio (ACR): **A** the impact of EVs on the ACR for fixed values of IL-9 (quintiles). There was a significant interaction between the variables (*p* = 0.0143); and **B** three-dimensional (3D) representation of the relationships between IL-9, EVs and ACR. All values were log-transformed for these analyses

The correlations between IL-9 and ACR (*r* = −0.26, *p* = 0.073) and between EV and ACR (*r* = 0.20, *p* = 0.16) were more modest and did not reach statistical significance (Table 3). However, there was a significant interaction between IL-9, EV and ACR (*p* = 0.0143). As shown in Fig. 2A, the relationship between EV and ACR is dependent on the IL-9 levels such that the steepness and direction of the relationship varies depending on the IL-9 percentile (10th to 90th) in the cohort. The 3-*D* plot in Fig. 2B is a visual representation of the interaction between these three variables.

**Table 3:** Spearman correlations coefficients and the interaction terms for three variable analyses with interleukin-9

Spearman correlations		
Variables	Correlation	*P* value
IL-9 and ACR	− 0.26011	0.0943
*Podocyte extracellular vesicle analysis*		
Podocyte EV and IL-9	− 0.55692	0.0004
Podocyte EV and ACR	0.20433	0.1629
*Interaction term*		
IL-9, podocyte EV, ACR	0.0143	
*VEGF analysis*		
VEGF and IL-9	0.71701	0.0004
VEGF and ACR	− 0.22874	0.1258
*Interaction term*		
IL-9, VEGF, ACR	0.0083	
*TNFα analysis*		
TNFα and IL-9	0.55391	0.0004
TNFα and ACR	− 0.23531	0.1258
*Interaction term*		
IL-9, TNFα, ACR	0.0231	
*IL-6 analysis*		
IL-6 and IL-9	0.46874	0.0023
IL-6 and ACR	− 0.09641	0.5099
*Interaction term*		
IL-9, IL-6, ACR	0.0178	

*IL-9* interleukin-9, *ACR* albumin/creatinine ratio, *EV* extracellular vesicle, *VEGF* vascular endothelial growth factor, *TNFα* tumor necrosis factor alpha, *IL-6* interleukin-6

### Spearman correlation coefficients relating urinary IL-9, urinary VEGF and ACR

**Fig. 3:**
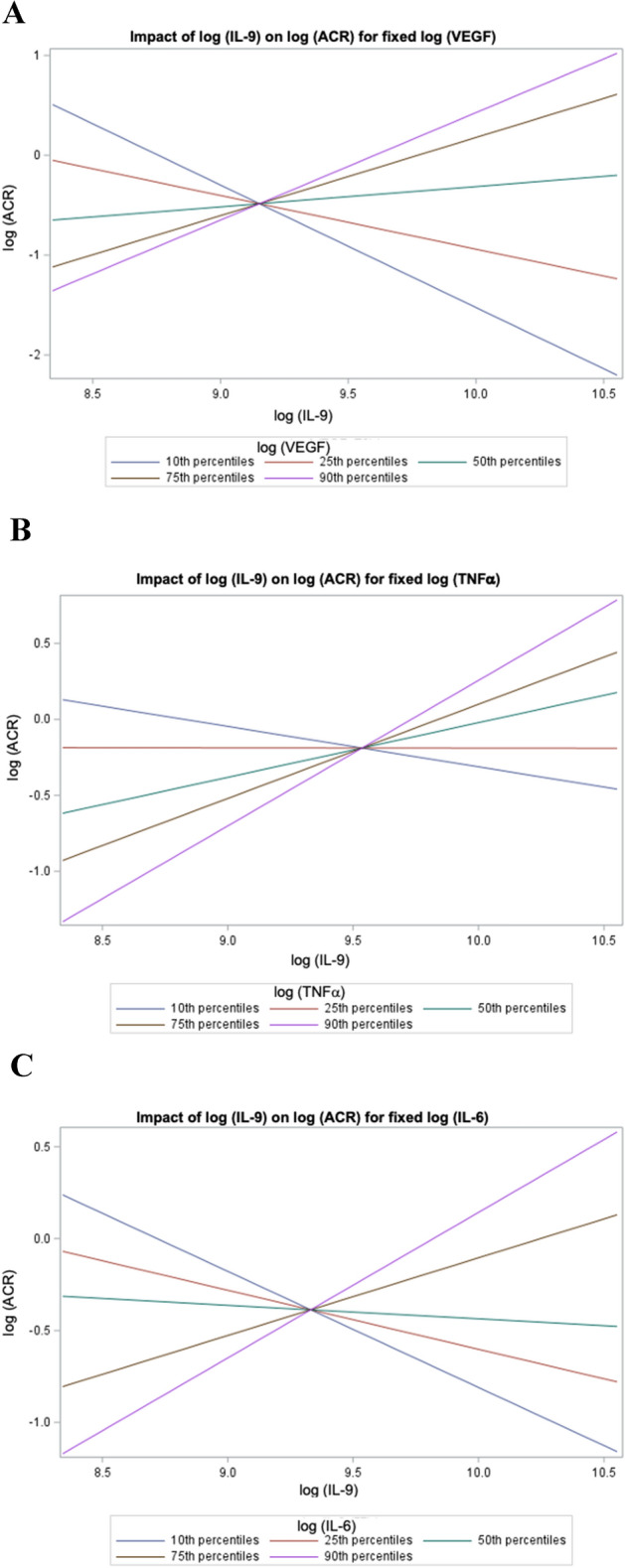
Interactions between interleukin-9 (IL-9), cytokines implicated in diabetic nephropathy including vascular endothelial growth factor (VEGF), tumor necrosis factor alpha (TNFα), interluekin-6 (IL-6) and the albumin/creatinine ratio (ACR) **A** the impact of IL-9 on the ACR for fixed values of VEGF (quintiles). There was a significant interaction between the variables (*p* = 0.0083); **B** the impact of IL-9 on the ACR for fixed values of TNFα (quintiles). There was a significant interaction between the variables (*p* = 0.0231); **C** the impact of IL-9 on the ACR for fixed values of IL-6 (quintiles). There was a significant interaction between the variables (*p* = 0.0178); all values were log-transformed for these analyses

There was a strong positive correlation between IL-9 and VEGF (*r* = 0.72, *p* < 0.001) such that higher values of IL-9 were associated with higher values of VEGF (Table 3). The modest correlation between VEGF and ACR (*r* = − 0.23, *p* = 0.13) did not reach statistical significance. There was, however, a significant interaction between IL-9, VEGF and ACR (*p* = 0.0083). As shown in Fig. 3A, the relationship between IL-9 and ACR is dependent on the VEGF levels such that the steepness and direction of the relationship varies depending on the VEGF percentile (10th to 90th).

### Spearman correlation coefficients relating urinary IL-9, urinary TNFα and ACR

There was a positive correlation between IL-9 and TNFα (*r* = 0.55, *p* < 0.001), but the correlation between TNFα and ACR (*r* = −0.24, *p* = 0.13) did not reach statistical significance (Table 3). There was a significant interaction between IL-9, TNFα and ACR (*p* = 0.0231). As shown in Fig. 3B, the relationship between IL-9 and ACR is also dependent on the TNFα levels. Again, the steepness and direction of the relationship varies depending on the TNFα percentile (10th to 90th).

### Spearman correlation coefficients relating urinary IL-9, urinary IL-6 and ACR

There was a positive correlation between IL-9 and IL-6 (*r* = 0.47, *p* = 0.002), but the correlation between IL-6 and ACR (*r* = − 0.10, *p* = 0.51) did not reach statistical significance (Table 3). There was a significant interaction between IL-9, IL-6 and ACR (*p* = 0.018). As shown in Fig. 3C, the relationship between IL-9 and ACR is also dependent on the IL-6 levels. The steepness and direction of the relationship varies depending on the IL-6 percentile (10th to 90th).

## Discussion

In this study, we sought to characterize relationships between IL-9 and early podocyte injury in youth with T1D. The rationale was based on recent studies that revealed a protective effect of IL-9 on glomerular podocyte injury in experimental kidney disease. [6] Our first major observation was that there was a negative correlation between urinary IL-9 and podocyte-derived EVs, a relationship that was similar in females and males. Although correlation does not establish causation, the current study is the first to relate urinary IL-9 to measures of glomerular podocyte injury in humans with T1D, and the finding is consistent with the hypothesis that IL-9 may limit podocyte injury in diabetic subjects, which is reflected by the release of EVs into the urine.

We have reported that urinary IL-9 levels are higher in adolescents with T1D compared to healthy age-matched subjects [23], but there is a paucity of data on urinary levels of IL-9 in diabetes. Vasanthakumar and coworkers reported that serum levels of IL-9 were lower in adults with type 2 diabetes and normal kidney function compared to healthy subjects [24]. They did find that IL-9 levels increased in participants with diabetic kidney disease [24]. Although IL-9 may limit podocyte injury in early diabetic kidney disease, its role in advanced disease is less clear.

Hyperglycemia, hyperfiltration and hypertension are risk factors for the onset and progression of diabetic nephropathy [25–28]. As part of our exploratory analysis, we therefore sought to relate IL-9 to glycemia (using both the blood glucose level at the time of urine collection and HbA1C), eGFR and blood pressure. There were negative correlations seen between urinary IL-9 with both HbA1C and BG at the time of urine collection. We similarly found lower values of IL-9 were associated with higher values of eGFR. These correlations support the hypothesis that IL-9 is reno-protective. In contrast, IL-9 was positively correlated with SBP but not with DBP. It is important to note that few of the study participants were hypertensive.

Our next major observation involved a functional correlate of podocyte injury, ACR. We discovered that there was a significant interaction between IL-9, podocyte-derived EVs and ACR. At low levels of urinary IL-9, there is a positive relationship between EVs and ACR, suggesting that as podocyte injury worsens, reflected by increasing EVs, there is a rise in the ACR. As IL-9 levels (represented as quintiles in Fig. 2A) rise, the association between EVs and ACR flattens, suggesting that urinary levels of IL-9 may change the relationship between podocyte injury reflected by EVs and ACR.

IL-9 was also strongly correlated with VEGF, TNFα and IL-6 (Table 2) all of which have all been previously implicated in diabetic nephropathy [16–18]. VEGF is primarily produced by podocytes and plays a critical role in maintenance of the fenestrated endothelium in the glomerular basement membrane [29]. IL-9 may limit podocyte injury and accordingly preserve VEGF synthesis. Members of the TNFα superfamily have been identified as biomarkers of kidney risk in several clinical studies of both T1D and type 2 diabetes [18, 30]. IL-6 expression is increased in diabetic nephropathy, and levels correlate with glomerular basement membrane thickening [17, 31]. Interestingly, both TNFα and IL-6 regulate IL-9 expression, which may explain the positive correlation [6, 32, 33].

ACR is a functional outcome of podocyte injury, and we found that there were significant interactions between IL-9 with VEGF, TNFα, and IL-6 and ACR (Table 3). The direction of the relationship between IL-9 and ACR was dependent on each of these cytokines (Table 3), and each cytokine exhibited a similar pattern. At low levels of VEGF, TNFα and IL-6 (10th percentile line in Fig. 3A, B, C), there is a negative relationship between IL-9 and ACR. At high levels (90th percentile line in Fig. 3A, B, C), there is a positive relationship between IL-9 and ACR. This suggests that kidney inflammation impacts the protective effect of IL-9 on podocyte function.

The strengths of this study include evaluation of a well-characterized cohort of adolescents with T1D with standardized collections and analysis of bio-specimens. However, there are also some important limitations. First, we did not identify the source of IL-9 in the urine. IL-9 is primarily produced by T helper cells recruited by IL-6 [34, 35] and by *T*-regulatory cells [36]. Animal models of diabetic nephropathy have shown helper *T* cells (CD4 +), cytotoxic (CD8 +) T cells and small numbers of *T*-regulatory cells in kidney [34, 37]. As inflammation in the kidney increases, infiltration of *T* cells may result in increased levels of IL-9. Second, the data are cross-sectional and therefore the implications of our findings with respect to the progression of ACR and declining GFR are unknown. Establishing an IL-9 threshold for high-risk patients could make it a clinically useful biomarker and establish its role as a potential treatment target. Long-term studies of larger cohorts will be required to address the limitation of our current study. Thirdly, the analyses are limited to correlations and interactions and do not establish causation. In vitro studies of cultured podocytes and studies of IL-9 gene deletion in murine models of diabetes can address these limitations. Finally, validation in other populations of TID will strengthen our conclusions and allow for generalizability.

## Conclusions

In summary, there is a strong relationship between urinary IL-9 and measures of glomerular podocyte injury (the release of EVs) in youth with T1D. Complex interactions between IL-9, cytokines and ACR support the hypothesis that inflammation is an important determinant of both glomerular injury and function in the diabetic kidney.

## Data Availability

The datasets used and/or analyzed during the current study are available from the corresponding authors on reasonable request.

## Abbreviations

BG: Blood glucose
VEGF: Vascular endothelial growth factor
ACR: Albumin/creatinine ratio
EV: Extracellular vesicle
SBP: Systolic blood pressure
DBP: Diastolic blood pressure

## References

[1] Goswami R, Kaplan MH (2011). A Brief History of IL-9. J Immunol.

[2] Chakraborty S, Kubatzky KF, Mitra D (2019). An update on interleukin-9: from its cellular source and signal transduction to its role in immunopathogenesis. Int J Mol Sci.

[3] Ouyang H, Shi Y, Liu Z, Feng S, Li L, Su N (2013). Increased Interleukin-9 and CD4+IL-9+ T cells in patients with systemic lupus erythematosus. Mol Med Rep.

[4] Hoppenot D, Malakauskas K, Lavinskiene S, Bajoriuniene I, Kalinauskaite V, Sakalauskas R (2015). Peripheral blood Th9 cells and eosinophil apoptosis in asthma patients. Med.

[5] Gregersen I, Skjelland M, Holm S, Holven KB, Krogh-Sørensen K, Russell D (2013). Increased systemic and local interleukin 9 levels in patients with carotid and coronary atherosclerosis. PLoS ONE.

[6] Xiong T, Attar M, Gnirck AC, Wunderlich M, Becker M, Rickassel C (2020). Interleukin-9 protects from early podocyte injury and progressive glomerulosclerosis in Adriamycin induced nephropathy. Kidney Int.

[7] Fioretto P, Mauer M (2007). Histopathology of diabetic nephropathy. Semin Nephrol.

[8] Toyoda M, Najafian B, Kim Y (2007). Podocyte detachment and reduced glomerular capillary endothelial fenestration in human type 1 diabetic nephropathy. Diabetes.

[9] Susztak K, Raff AC, Schiffer M, Böttinger EP (2006). Glucose-induced reactive oxygen species cause apoptosis of podocytes and podocyte depletion at the onset of diabetic nephropathy. Diabetes.

[10] Shah R, Patel T, Freedman JE (2018). Circulating extracellular vesicles in human disease. N Engl J Med.

[11] Medeiros T, Myette RL, Almeida JR, Silva AA, Burger D (2020). Extracellular vesicles: cell-derived biomarkers of glomerular and tubular injury. Cell Physiol Biochem.

[12] Erdbrügger U, Le TH (2016). Extracellular vesicles in renal diseases: more than novel biomarkers?. J Am Soc Nephrol.

[13] Burger D, Thibodeau JF, Holterman CE, Burns KD, Touyz RM, Kennedy CRJ (2014). Urinary podocyte microparticles identify prealbuminuric diabetic glomerular injury. J Am Soc Nephrol.

[14] Lytvyn Y, Xiao F, Kennedy CRJ, Perkins BA, Reich HN, Scholey JW (2017). Assessment of urinary microparticles in normotensive patients with T1D. Diabetologia.

[15] Sullivan KM, Scholey J, Moineddin R, Sochett E, Wicklow B, Elia Y (2021). Urinary podocyte-derived microparticles in youth with type 1 and type 2 diabetes. Diabetologia.

[16] Sivaskandarajah GA, Jeansson M, Maezawa Y, Eremina V, Baelde HJ, Quaggin SE (2012). Vegfa protects the glomerular microvasculature in diabetes. Diabetes.

[17] Suzuki D, Miyazaki M, Naka R, Koji T, Yagame M, Jinde K, Endo M, Nomoto Y, Sakai H (1995). In situ hybridization of interleukin 6 in diabetic nephropathy. Diabetes.

[18] Purohit S, Sharma A, Zhi W, Bai S, Hopkins D, Steed L (2018). Proteins of TNF-α and IL6 pathways are elevated in serum of type-1 diabetes patients with microalbuminuria. Front Immunol.

[19] Har RLH, Reich HN, Scholey JW, Daneman D, Dunger DB, Moineddin R (2014). The urinary cytokine/chemokine signature of renal hyperfiltration in adolescents with T1D. PLoS ONE.

[20] Cherney DZI, Scholey JW, Daneman D, Dunger DB, Dalton RN, Moineddin R (2012). Urinary markers of renal inflammation in adolescents with type1 diabetes mellitus and normoalbuminuria. Diabet Med.

[21] Diabetes Canada Clinical Practice Guidelines Expert Committee (2018). Diabetes Canada 2018 clinical practice guidelines for the prevention and management of diabetes in Canada. Can J Diabet.

[22] Flynn JT, Falkner BE (2017). New clinical practice guideline for the management of high blood pressure in children and adolescents. Hypertension.

[23] De Melo EN, Deda L, Har R, Reich HN, Scholey JW, Daneman D (2016). The urinary inflammatory profile in gluten free diet-Adherent adolescents with T1D and celiac disease. J Diabet Complications.

[24] Vasanthakumar R, Mohan V, Anand G, Deepa M, Babu S, Aravindhan V (2015). Serum IL-9, IL-17, and TGF-β levels in subjects with diabetic kidney disease (CURES-134). Cytokine.

[25] The Microalbuminuria Collaborative Study Group (1999). Predictors of the development of microalbuminuria in patients with Type 1 diabetes mellitus: a seven-year prospective study. Diabet Med.

[26] The Diabetes Control and Complications Trial Research Group (1993). The effect of intensive treatment of diabetes on the development and progression of long-term complications in insulin-dependent diabetes mellitus. N Engl J Med.

[27] Dahlquist G, Stattin EL, Rudberg S (2001). Urinary albumin excretion rate and glomerular filtration rate in the prediction of diabetic nephropathy: a long-term follow-up study of childhood onset type-1 diabetic patients. Nephrol Dial Transpl.

[28] Lewis JB, Berl T, Bain RP, Rohde RD, Lewis EJ (1999). Effect of intensive blood pressure control on the course of type 1 diabetic nephropathy. Am J Kidney Dis.

[29] Majumder S, Advani A (2017). VEGF and the diabetic kidney: more than too much of a good thing. J Diabet Complicat.

[30] Fernández-Real JM, Vendrell J, García I, Ricart W, Valles M (2012). Structural damage in diabetic nephropathy is associated with TNF-α system activity. Acta Diabetol.

[31] Dalla Vestra M, Mussap M, Gallina P, Bruseghin M, Cernigoi AM, Saller A, Plebani M, Fioretto P (2005). Acute-phase markers of inflammation and glomerular structure in patients with type 2 diabetes. J Am Soc Nephrol.

[32] Jiang Y, Chen J, Bi E, Zhao Y, Qin T, Wang Y (2019). TNF-α enhances Th9 cell differentiation and antitumor immunity via TNFR2-dependent pathways. J Immunother Cancer.

[33] Schütze N, Trojandt S, Kuhn S, Tomm JM, von Bergen M, Simon JC (2016). Allergen-Induced IL-6 Regulates IL-9/IL-17A Balance in CD4 + T Cells in Allergic Airway Inflammation. J Immunol.

[34] Moon JY, Jeong KH, Lee TW, Ihm CG, Lim SJ, Lee SH (2012). Aberrant recruitment and activation of T cells in diabetic nephropathy. Am J Nephrol.

[35] Kimura A, Kishimoto T (2010). IL-6: regulator of Treg/Th17 balance. Eur J Immunol.

[36] Eller K, Wolf D, Huber JM, Metz M, Mayer G, McKenzie ANJ (2011). IL-9 production by regulatory T cells recruits mast cells that are essential for regulatory T cell-induced immune suppression. J Immunol.

[37] Mensah-Brown EPK, Obineche EN, Galadari S, Chandranath E, Shahin A, Ahmed I (2005). Streptozotocin-induced diabetic nephropathy in rats: the role of inflammatory cytokines. Cytokine.

